# Tegaserod Maleate Inhibits Esophageal Squamous Cell Carcinoma Proliferation by Suppressing the Peroxisome Pathway

**DOI:** 10.3389/fonc.2021.683241

**Published:** 2021-08-04

**Authors:** Xiangyu Wu, Zitong Wang, Yanan Jiang, Hao Zhou, Ang Li, Yaxing Wei, Zhuo Bao, Donghao Wang, Jimin Zhao, Xinhuan Chen, Yaping Guo, Zigang Dong, Kangdong Liu

**Affiliations:** ^1^Pathophysiology Department, School of Basic Medical Sciences, Zhengzhou University, Zhengzhou, China; ^2^China-US (Henan) Hormel Cancer Institute, Zhengzhou, China; ^3^Provincial Cooperative Innovation Center for Cancer Chemoprevention, Zhengzhou University, Zhengzhou, China; ^4^State Key Laboratory of Esophageal Cancer Prevention and Treatment, Zhengzhou, China; ^5^Cancer Chemoprevention International Collaboration Laboratory, Zhengzhou, China

**Keywords:** esophageal squamous cell carcinoma, tegaserod maleate, proteome, peroxisome, peroxisome membrane protein 11B, peroxisome membrane protein 13, patient-derived xenograft

## Abstract

Esophageal squamous cell carcinoma (ESCC) and esophageal adenocarcinoma (EAC) are the two major types of esophageal cancer (EC). ESCC accounts for 90% of EC. Recurrence after primary treatment is the main reason for poor survival. Therefore, recurrence prevention is a promising strategy for extending the 5-year survival rate. Here, we found tegaserod maleate could inhibit ESCC proliferation both *in vivo* and *in vitro*. Proteomics analysis revealed that tegaserod maleate suppressed the peroxisome signaling pathway, in which the key molecules peroxisome membrane protein 11B (PEX11B) and peroxisome membrane protein 13 (PEX13) were downregulated. The immunofluorescence, catalase activity assay, and reactive oxygen species (ROS) confirmed that downregulation of these proteins was related to impaired peroxisome function. Furthermore, we found that PEX11B and PEX13 were highly expressed in ESCC, and knockout of PEX11B and PEX13 further demonstrated the antitumor effect of tegaserod maleate. Importantly, tegaserod maleate repressed ESCC tumor growth in a patient-derived xenograft (PDX) model *in vivo*. Our findings conclusively demonstrated that tegaserod maleate inhibits the proliferation of ESCC by suppressing the peroxisome pathway.

## Introduction

Esophageal cancer (EC) is the seventh most common cancer worldwide and the sixth most common cause of cancer-related deaths (1). Esophageal squamous cell carcinoma (ESCC)—the main histological subtype of EC—accounts for about 90% of all cases, especially in areas with high incidence, such as Asia (2). At present, the therapeutic approaches including chemotherapy, radiotherapy, and surgical resection are the mainstream treatments for EC patients. However, recurrence after primary treatment is common reason leading to a poor prognosis (3). Chemoprevention recurrence of ESCC is a promising strategy for improving patient survival.

Repurposing of existing drugs is a time-saving means of developing chemopreventive drugs with higher efficacy and fewer side effects (4). Abundant evidence has shown that FDA-approved non-antitumor drugs, such as antibiotics, anti-inflammatory drugs, lipid-lowering drugs, and sulfonylureas, have shown anti-tumor effects in a variety of cancers (5).

Tegaserod maleate, a 5-hydroxytryptamine 4-receptor partial agonist, was approved for treatment of constipation-type irritable bowel syndrome or functional constipation (6). In recent years, tegaserod maleate has been reported to inhibit the growth of cancer cells in cancer research, but the mechanism of tegaserod maleate inhibition of tumor proliferation has not been studied.

Peroxisomes are organelles involved in the immune system, lipid metabolism, cardiovascular health, brain development, and nerve function (7). Children born with hereditary peroxisomal diseases, such as Zellweger syndrome, have severe developmental and neurological defects (8). Catalase—the marker enzyme of peroxisomes—accounts for about 40% of the total enzyme content of peroxisomes (9). Peroxisomes are important for maintaining cell homeostasis by regulating reactive oxygen species (ROS) mainly through scavenging system (10). Peroxisome membrane protein 11B (PEX11B) and peroxisome membrane protein 13 (PEX13) are proteins necessary for peroxisome biogenesis (11).

In the present study, through drug screening, we found that tegaserod maleate effectively inhibits ESCC proliferation. We demonstrated tegaserod maleate could inhibit ESCC proliferation both *in vivo* and *in vitro*. Molecular study indicated peroxisome signaling pathway was suppressed by tegaserod maleate. This study aimed to highlight the potential clinical use of tegaserod maleate in ESCC chemoprevention.

## Materials and Methods

### Reagents and Antibodies

Tegaserod maleate was purchased from Selleck Chemicals LLC (Houston, TX, USA). After dissolving in dimethyl sulfoxide to a final concentration of 50 mM, tegaserod maleate was aliquoted and stored at −80°C. Antibodies to detect PEX11B and Ki67 were purchased from Abcam (Cambridge, UK). PEX6 for western blotting was from Signalway Antibody (Pearland, TX, USA). Antibodies to detect PEX13 and PMP70 were from Santa Cruz Biotechnology (Santa Cruz, CA, USA). GAPDH was from Goodhere Biological Technology Co. Ltd. (Hangzhou, Zhejiang, China).

### Cell Culture

Human ESCC cell lines KYSE150 and KYSE450 were obtained from the Chinese Academy of Sciences (Beijing, China). The Shantou human esophageal epithelium (SHEE) cell line was gifted by Shantou University (Guangdong, China). All cell lines were maintained in RPMI-1640 medium or Dulbecco’s modified Eagle medium supplemented with 10% fetal bovine serum, and cultured at 37°C in a humidified incubator with 5% CO_2_.

### Cytotoxicity Assay

ESCC cells (KYSE150 cells: 8×10^3^ cells/well; KYSE450 cells: 1.2×10^3^ cells/well) were seeded in 96-well plates and cultured with corresponding medium overnight. After incubation for 16 h, cells were treated with tegaserod maleate (0, 3.125, 6.25, 12.5, 25, and 50 μM) for 24 and 48 h. Cells were fixed for 30 min with 4% paraformaldehyde in phosphate buffered saline (PBS) and then stained with 4′,6-diamidino-2-phenylindole (DAPI) at 37°C for 20 min. Cells were counted by using IN Cell Analyzer 6000 software (General Electric Company, GE, USA). The half-maximal (median) inhibitory concentration (IC_50_) was evaluated by SPSS statistical software, version 21 (IBM Corp., Armonk, NY, USA). Experiments were performed independently three times.

### Cell Proliferation Assay

To estimate viability, KYSE150 (2×10^3^ cells/per well) and KYSE450 (4×10^3^ cells/per well) cells were seeded into 96-well plates and incubated for 16 h. Cells were exposed to different concentrations of tegaserod maleate (0, 0.25, 0.5, 1, and 2.5 μM) for 24, 48, 72, and 96 h. Cells were fixed by 4% paraformaldehyde for 30 min at 25°C. Then, DAPI was used to stain cells at 37°C for 20 min, and cells were counted using IN Cell Analyzer 6000 software. Experiments were performed independently three times in triplicate.

### Soft Agar Assay

To assess anchorage-independent cell growth, cells (8×10^3^ cells/well) suspended in medium were added to 1.25% agar with different concentrations of tegaserod maleate (0, 0.25, 0.5, 1, and 2.5 μM). The cultures were maintained at 37°C in a 5% CO_2_ incubator for 2 weeks and then colonies were counted using the IN Cell Analyzer 6000 software. Experiments were performed independently three times.

### Colony Formation Assay

KYSE150 (200 cells/well) and KYSE450 (500 cells/well) cells were seeded in 6-well plates. Cells were treated with tegaserod maleate (0, 0.25, 0.5, 1, and 2.5 μM) for a designated time and fixed 14 days later with 4% paraformaldehyde for 30 min, and stained with 0.1% crystal violet for 3 min. Colonies containing more than 50 cells were photographed by phone camera, then the number of colonies was measured.

### Western Blotting

After cells were treated with different concentrations of tegaserod maleate (0, 0.25, 0.5, 1, and 2.5 μM) for 24 h, they were harvested and lysed by radioimmunoprecipitation assay buffer to obtain protein samples. Protein concentration was determined using a bicinchoninic acid protein assay kit (BCA Protein Assay Kit, Beyotime Biotechnology, Shanghai, China). The protein samples were resolved by sodium dodecyl sulfate-PAGE and then transferred onto polyvinylidene fluoride membranes. The membranes were blocked with 5% bovine serum albumin (BSA) or 5% skim milk. Next, the membranes were incubated with primary antibodies overnight at 4°C, followed by incubation with specific secondary antibodies for 2 h at room temperature. Protein bands were visualized using the enhanced chemiluminescence (ECL) detection reagent (Dalian Meilun Biotechnology Co., Ltd, Dalian, China).

### Immunofluorescence

KYSE150 and KYSE450 cells were added to 12-well plates at 1.5×10^5^ cells/well and incubated overnight. Cells were treated with tegaserod maleate (0, 0.25, 0.5, 1, and 2.5 μM) for 24 h, fixed with 4% paraformaldehyde, and placed at −20°C overnight. The next day, the cells were permeabilized in 0.5% Triton X‐100/PBS and blocked with blocking buffer (1% BSA-PBS-Tween) for 1.5 h at room temperature. The coverslips or slides were incubated with primary antibodies at 4°C overnight and then with fluorescent secondary antibody for 1.5 h at room temperature. Nuclei were stained using DAPI. Cells or slides were mounted with antifluorescence quencher. Fluorescence images were obtained with IN Cell Analyzer 6000 software and analyzed quantitatively.

### Catalase Activity Assay

The CAT activity in the peroxisomes was detected using a catalase assay kit (Beyotime Biotechnology) according to manufacturer’s instructions. Briefly, under catalysis of peroxidase, residual hydrogen peroxide produces a red product, *N*-4-antipyryl-3-chloro-5-sulfonate-*p*-benzoquinonemonoimine, that has an absorption maximum at 520 nm. CAT activity was calculated from the assay results.

### Reactive Oxygen Species Assay

Production of ROS was monitored by using a Reactive Oxygen Species Assay Kit (Beyotime Biotechnology) according to the manufacturer’s protocol. KYSE150 and KYSE450 cells treated with tegaserod maleate (0, 0.25, 0.5, 1, and 2.5 μM) were incubated with 10 mM 2,7-dichlorofluorescein diacetate (DCFH-DA) at 37°C in a humidified incubator with 5% CO_2_ for 20 min. Fluorescence images and DCF fluorescence intensities were analyzed using the IN Cell Analyzer 6000.

### Tissue Microarray

The tissue microarray included tumors and adjacent normal tissues from 114 cases of ESCC (Shanghai Xinchao Biotechnology Co. Ltd., Shanghai, China). PEX13 and PEX11B were detected according to standard immunohistochemical methods, and their expression was evaluated and scored on the basis of staining intensity and area. The intensity was scored as follows: 0, negative; 1, weak; 2, moderate; 3, strong. All tissues were photographed using a microscope camera and analyzed by TissueFAXS Viewer software program (TissueGnostics, Austria).

### CRISPR/Cas9 Knockout Cell Lines

PEX11B and PEX13 were deleted in ESCC cells by using the CRISPR/Cas9 system. Following the manufacturer’s suggested protocols, viral vectors and packaging vectors were transfected into 293T cells by using Jet Primer (ThermoFisher Scientific, Waltham, MA, USA). After transfection, the medium was changed at 4 h, and cells were cultured for 24 and 48 h. Viral particles were harvested by filtration using a 0.22-mm syringe filter, and then combined with 8 μg/mL polybrene and used to infect KYSE150 cells. KYSE150 cells were selected with 2 μg/mL puromycin for 72 h. Knockout efficacy was determined by western blotting. The oligonucleotide sequences of PEX11B and PEX13 single guide (sg) RNA were designed from an online CRISPR/Cas9 tool (CHOPCHOP: https://chopchop.cbu.uib.no/) (12) and were listed as **Table 1**.

**Table 1:** The oligonucleotide sequences of PEX11B and PEX13 single guide (sg) RNA.

Gene Name	Primer sequences 5′‐3′
PEX11B#3	F: CACCGCTTTCTTCCAAGGCTCAGG
	R: AAACCCTGAGCCTTGGAAGAAAGC
PEX11B#4	F: CACCGGGCATCTGCTGAGTTACCC
	R: AAACGGGTAACTCAGCAGATGCCC
PEX13#3	F: CACCGATCTTTACAGACGGCTACAG
	R: AAACCTGTAGCCGTCTGTAAAGATC
PEX13#4	F: CACCGAAATGAGGTAAGGACCACCA
	R: AAACTGGTGGTCCTTACCTCATTTC

F = Forward, R = Reverse.

### PDX Esophageal Cancer Mouse Model

To examine the effect of tegaserod maleate on ESCC PDX tumor growth, severe combined immunodeficiency (SCID) mice (6 to 8 weeks old; Vital River Labs, Beijing, China) were used. ESCC tissues (named LEG34) were cut into pieces and planted into the back of the neck of the mice. Mice were randomly divided into three groups (7 animals each) as follows: the vehicle group (normal saline group), 2 mg/kg tegaserod maleate group, and 10 mg/kg tegaserod maleate group. The tegaserod maleate or vehicle was orally administered once every day. Mice were monitored until the average tumor volume in the vehicle group reached a volume of 1000 mm^3^, at which time treatment was stopped and the tumor tissue was harvested. The tumor growth inhibition (TGI) rate was determined according to the calculation formula, as follows: TGI=100[1-(Treatment final volume-Treatment initial volume)/(Control final volume-Control initial volume). All animal assays were performed under the supervision of the Zhengzhou University Institutional Animal Care and Use Committee (Zhengzhou, Henan, China). All protocols were approved by the Research Ethics Committee of Zhengzhou University.

### Immunohistochemical Staining

Tumor tissues from mice were embedded in paraffin blocks for immunohistochemical staining and analysis. Tissues sections were deparaffinized and hydrated. Sections were incubated with 3% H_2_O_2_ for 10 min and then incubated at 4°C with primary antibodies overnight. Secondary antibody was added and incubated at 37°C for 15 min. Tissue sections were stained using diaminobenzidine and hematoxylin. Finally, sections were dehydrated and covered with glass slides. All tissue sections were photographed using a microscope camera and analyzed using TissueFAXS Viewer software program.

### Statistical Analyses

SPSS statistical software, version 21 (IBM Corp.) was used for all statistical analyses. The results were analyzed via Student’s *t*-test or one-way analysis of variance. Quantitative data were expressed as mean values ± standard deviations. Statistical significance was defined by a *p*-value < 0.05.

## Results

### Tegaserod Maleate Inhibited ESCC Cells Proliferation *In Vitro*

**Figure 1:**
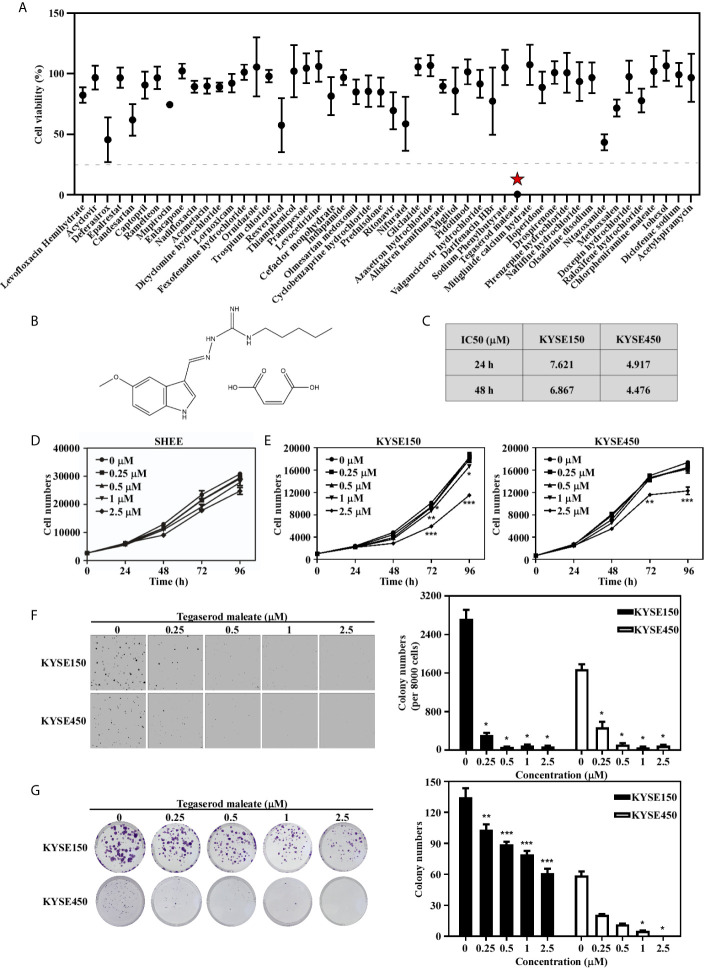
Tegaserod maleate inhibits ESCC cells proliferation *in vitro*. **(A)** A cell viability screening identified tegaserod maleate has anti-esophageal cancer activity among the FDA approved compounds. **(B)** The chemical structure of tegaserod maleate. **(C)** The different IC_50_ values of KYSE150, KYSE450 according to the cytotoxicity assay. **(D)** The effect of tegaserod maleate on immortalized human esophageal epithelial cell line SHEE cells viability. **(E)** KYSE150 cells (left panel) and KYSE450 cells (right panel) were treated with different concentrations of tegaserod maleate (0, 0.25, 0.5, 1.0 and 2.5 µM). Tegaserod maleate effectively suppresses anchorage-independent cell growth **(F)** and plate colony formation **(G)**. Left panel: the representative pictures of clones. Right panel: the statistic of colony numbers (n = 3). Data was shown with mean ± SD. (*p < 0.05, **p < 0.01, ***p < 0.001 vs. untreated control, n ≥ 3).

To identify drugs with anti-esophageal cancer activity, we screened FDA-approved drugs and found tegaserod maleate had an inhibitory effect and resulted in a cell viability rate of only 0.29% in KYSE450 cells at 50 μM (**Figure 1A**). The chemical structure of tegaserod maleate is shown in **Figure 1B**. Through a cytotoxicity assay (**Figure S1**), we found that the half-maximal inhibitory concentration (IC_50_) value in KYSE150 cells was 6.867 μM at 48 h and that in KYSE450 cells was 4.476 μM (**Figure 1C**). The IC_50_ in the immortalized human esophageal epithelial cell line SHEE was 9.688 μM (**Figure S2**). These results indicated that tegaserod maleate had a significant inhibitory effect on KYSE150 and KYSE450 cells (*p* < 0.05) but a less effect on SHEE cells (**Figure 1D**). We then performed a proliferation assay (**Figure 1E**), an anchor-independent cell growth assay (**Figure 1F**), and a colony formation assay (**Figure 1G**). We found that tegaserod maleate significantly inhibited anchorage-dependent growth and anchorage-independent growth of ESCC cells in a dose-dependent manner. Together, these results confirmed that tegaserod maleate could inhibit the proliferation of ESCC cells *in vitro*.

### Tegaserod Maleate Treatment Changed Protein Constituents of KYSE150 Cells

**Figure 2:**
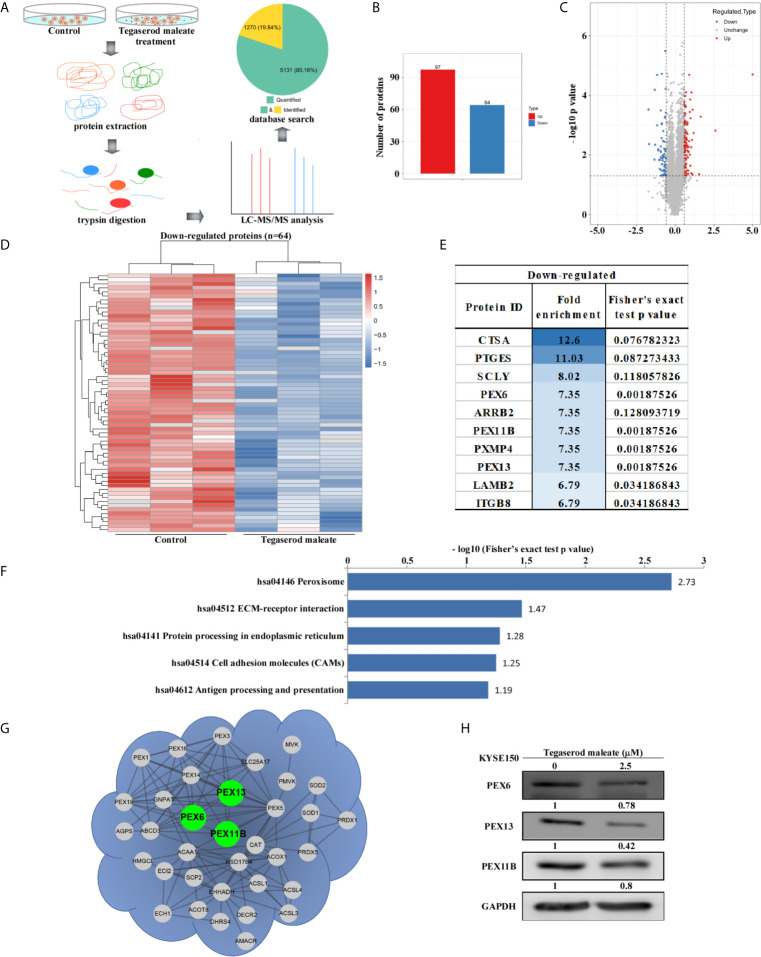
Proteomic analysis suggests that tegaserod maleate inhibits ESCC cells *via the* peroxisome pathway. **(A)** After tegaserod maleate treatment (2.5 μM) for 24 h, the general procedure of the experiment is to illustrate the proteomics process of KYSE150 cells. **(B)** A total of 161 differentially expressed proteins were detected. **(C)** Volcano plot shows that 161 proteins have undergone significant changes (fold change > 1.5, *p* < 0.05). Red dots represent upregulated proteins, and blue dots represent downregulated proteins. **(D)** The heatmap of down-regulated proteins after normalization (n = 64). **(E)** The top 10 differentially expressed proteins were ranked according to the enrichment score among the down-regulated proteins. **(F)** A bar chart representing the KEGG pathway enrichment analysis of down-regulated proteins enrichment that was altered due to tegaserod maleate treatment. **(G)** The proteins involved in peroxisome pathway identified after tegaserod maleate treatment were mapped to the network through the STRING database (confidence score > 0.7), in which the points are proteins. **(H)** Western blotting of tegaserod maleate-treated KYSE150 cells verifies the above results.

To explore the mechanism by which tegaserod maleate inhibits ESCC proliferation *in vitro*, we performed a proteomics analysis. Cells were harvested for mass spectrometry analysis after a 24-h treatment with tegaserod maleate. The process is illustrated in **Figure 2A**. In total, compared with an untreated control group, 6401 differential proteins were detected, of which 5131 had quantitative information. After normalization, 161 differentially expressed proteins were detected (**Figure 2B**). Of these proteins, 97 were upregulated and 64 were downregulated (fold change >1.5, *p* < 0.05) (**Figure 2C**). The quantitative values of all differentially expressed proteins were normalized using heat maps for downregulated proteins (**Figure 2D**) and upregulated proteins (**Figure S3**). We ranked the downregulated differentially expressed proteins and displayed the top 10 in accordance with the enrichment score (**Figure 2E**). The downregulated proteins were enriched in the Kyoto Encyclopedia of Genes and Genomes (KEGG) database. The top five pathways were displayed from small to large according to Fisher’s exact test *p*-value, and only the first two pathways were statistically significant (*p* < 0.05). The KEGG database showed that drug-altered proteins, including PEX6, PEX11B, PXMP4, and PEX13, were closely related to the peroxisome signaling pathway (**Figure 2F**). The spectra of these proteins were represented by the highest specific peptide score (**Figure S4**). With a confidence level >0.7 as the threshold, only three differentially expressed proteins, namely, PEX6, PEX11B, and PEX13 interacted in the peroxisome pathway, in accordance with data in the STRING database (**Figure 2G**). Western blotting showed that levels of PEX6, PEX11B, and PEX13 were decreased in KYSE150 cells treated with tegaserod maleate (**Figure 2H**). Moreover, using peroxisome integral membrane protein PMP70 as the marker protein of peroxisome, we found that PMP70 was downregulated in tegaserod maleate-treated KYSE150 cells through western blotting (**Figure S5A**). These findings indicated that peroxisomes and levels of peroxisome-related proteins were downregulated following tegaserod maleate treatment.

### Tegaserod Maleate Restricted Peroxisome Function in ESCC Cells

**Figure 3:**
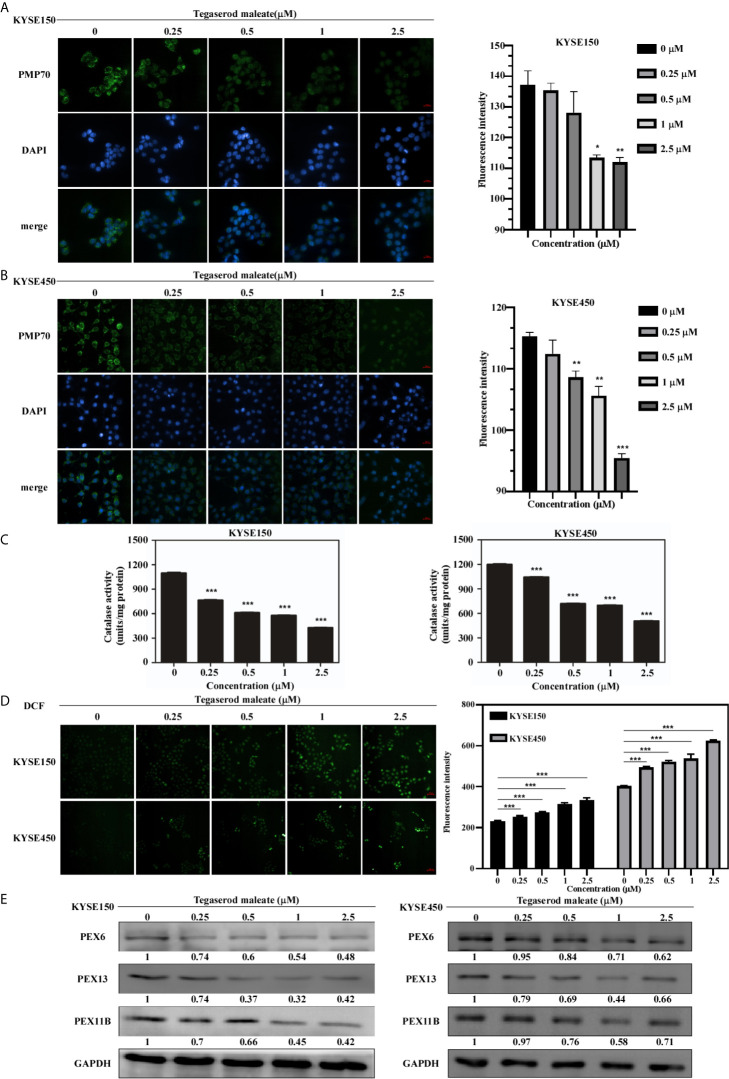
Tegaserod maleate inhibits the peroxisome function in ESCC cells by PEX6, PEX11B, and PEX13. Immunofluorescence results showed the downregulation of PMP70 in KYSE150 cells **(A)** and KYSE450 cells **(B)**. Left panel: the representative pictures of fluorescence (scale, 20 μm). Right panel: the statistics of fluorescence intensity. Data is shown with mean ± SD. (*p < 0.05, **p < 0.01, ***p < 0.001 *vs*. untreated control, n = 4). **(C)** With increasing concentration of tegaserod maleate (0, 0.25, 0.5, 1.0 and 2.5 μM), the catalase activity is decreased in KYSE150 cells and KYSE450 cells. **(D)** Dose-dependent effects of tegaserod maleate on cellular ROS levels of KYSE150 cells and KYSE450 cells, respectively. Left panel: representative pictures of fluorescence (scale, 60 μm). Right panel: statistics of fluorescence intensity. Data is shown with mean ± SD. (*p < 0.05, **p < 0.01, ***p < 0.001 *vs*. untreated control, n = 10). **(E)** Western blotting shows that the levels of PEX6, PEX11B and PEX13 are decreased after treatment with tegaserod maleate.

Peroxisomes play an important role in growth and cell balance (13). Therefore, we probed whether tegaserod maleate would affect peroxisome function. An immunofluorescence assay indicated that the fluorescence intensity of the peroxisome integral membrane protein PMP70 decreased by 18.4% in KYSE150 cells (**Figure 3A**) and by 17.2% in KYSE450 cells (**Figure 3B**) treated with 2.5 μM of tegaserod maleate. Similarly, we assayed the activity of catalase, a marker enzyme of peroxisomes, and showed that tegaserod maleate attenuated catalase activity in KYSE150 and KYSE450 cells. This inhibition showed concentration dependence (**Figure 3C**). Consistent with previous results, the level of ROS in ESCC cells increased after tegaserod maleate treatment, presumably because the peroxisomes with attenuated catalase activity were unable to eliminate intracellular hydrogen peroxide (**Figure 3D**). Additionally, we verified, by western blotting, that protein levels of PEX6, PEX11B, and PEX13 decreased with increasing tegaserod maleate concentration (**Figure 3E**). Collectively, these results indicated that tegaserod maleate downregulates peroxisome function.

### PEX11B and PEX13 Were Highly Expressed in ESCC

**Figure 4:**
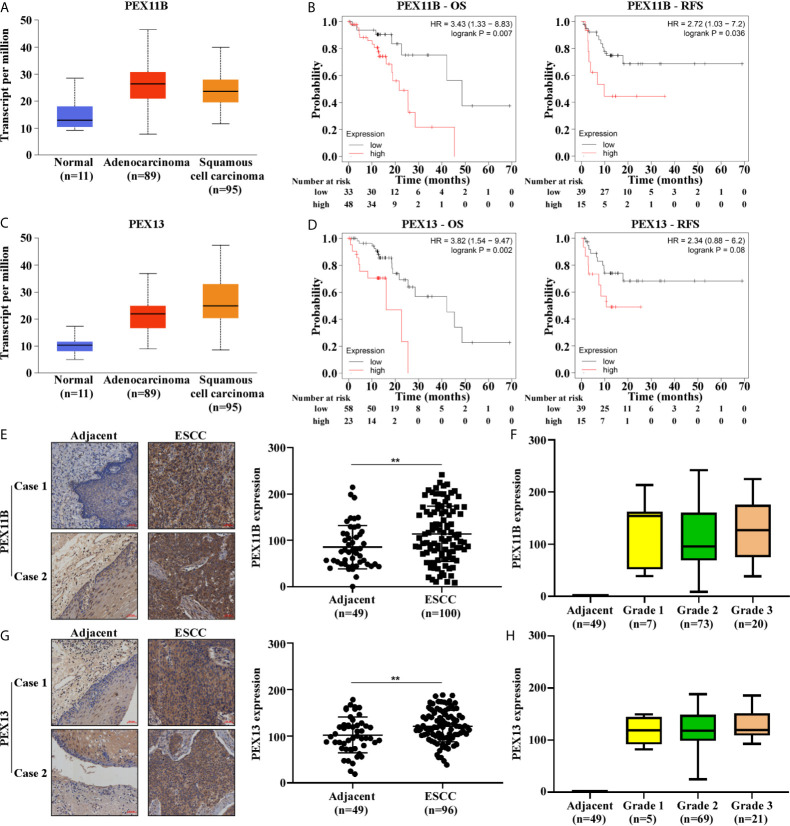
The expression of PEX11B and PEX13 in ESCC are higher and are closely related to the prognosis of ESCC. **(A)** The expression level of PEX11B in ESCC from the TCGA database. **(B)** Kaplan–Meier curves of OS and RFS in patients with PEX11B high expression. **(C)** The expression of PEX13 in UALCAN. **(D)** Kaplan–Meier survival was used to show OS and RFS in PEX13 high expression patients. **(E)** Tissue microarray shows high expression of PEX11B in ESCC (**p < 0.01). The image on the left is representative. **(F)** The tumor grade in PEX11B high expression cases. **(G)** Representative staining images from PEX13 high expression tissues (left), and the scatter plot of PEX13 high expression in ESCC (right) (**p < 0.01). **(H)** The pathology grade in PEX13 high expression patients.

PEX11B and PEX13 are important membrane components of peroxisomes: PEX11B participates in the elongation of peroxisomes (14) and PEX13 is involved in the transport of matrix enzymes (15). We searched for TCGA gene expression data in UALCAN (16) and found that PEX11B and PEX13 were upregulated in ESCC (**Figures 4A, C**). Subsequently, we used Kaplan Meier Plotter (17) to observe that high expression of PEX11B and PEX13 in ESCC patients predict poor overall survival (OS) and recurrence-free survival (RFS) (**Figures 4B, D**). On comparing expression of PEX11B and PEX13 between ESCC samples and normal tissues using a tissue microarray, PEX11B and PEX13 were found to be highly expressed in ESCC (**Figures 4E, G**) and correlated with clinicopathological grade (**Figures 4F, H**). In addition, we also examined the expression of PEX6, but TCGA database showed that PEX6 was not highly expressed in ESCC and was not associated with a poor prognosis (**Figure S6**).

### Inhibitory Effect of Tegaserod Maleate on ESCC Cells Are PEX11B and PEX13 Dependent

**Figure 5:**
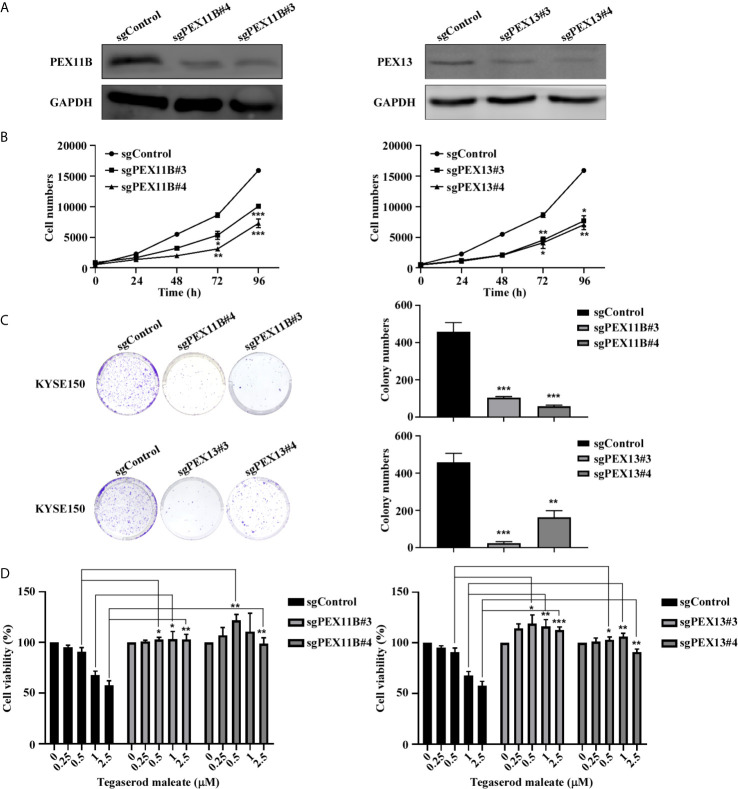
Tegaserod maleate inhibits KYSE150 cells proliferation depends on PEX11B and PEX13. **(A)** Western blotting verified the knockout efficiency of PEX11B and PEX13 in KYSE150 cells. **(B)** Cell growth was inhibited in KYSE150 cells after knocking out PEX11B and PEX13. **(C)** On the 7th day after cell seeding, detect the effect of sgPEX11B and sgPEX13 in KYSE150 cells plate colony formation compared with sgControl. Left panel: the representative pictures of clones. Right panel: the statistic of colony numbers (n = 3). **(D)** Treated with different concentrations of tegaserod maleate (0, 0.25, 0.5, 1.0 and 2.5 µM) for 96 h, PEX11B and PEX13 knockout KYSE150 cells were significantly less sensitive to tegaserod maleate than sgControl. Data was shown with mean ± SD. (*p < 0.05, **p < 0.01, ***p < 0.001 *vs*. untreated control, n ≥ 3).

We further explored the role of PEX11B and PEX13 in tumor suppression. First, we established KYSE150-stably transfected cell lines with a plasmid expressing sgRNA against PEX11B (sgPEX11B) and or a plasmid expressing sgRNA against PEX13 (sgPEX13) respectively and a control sequence (sgControl). We verified the knockout effect of PEX11B and PEX13 by western blotting, respectively (**Figure 5A**). Subsequently, we performed a cell proliferation assay (**Figure 5B**) and a colony formation assay (**Figure 5C**). The results showed that cell proliferation was significantly inhibited after silencing PEX11B or PEX13. On the basis of these results, we hypothesize whether the inhibitory effect of tegaserod maleate depends on the expression of PEX11B or PEX13. Therefore, we treated PEX11B and PEX13 knockout KYSE150 cells respectively with tegaserod maleate. After 96 h, cell viability indicated that knocking out PEX11B or PEX13 in KYSE150 cells were less sensitive to different concentrations of tegaserod maleate than sgControl group (**Figure 5D**). In summary, we concluded that the inhibitory effect of tegaserod maleate depended on PEX11B and PEX13 protein levels.

### Tegaserod Maleate Inhibited PDX Growth in Tumors *In Vivo*

**Figure 6:**
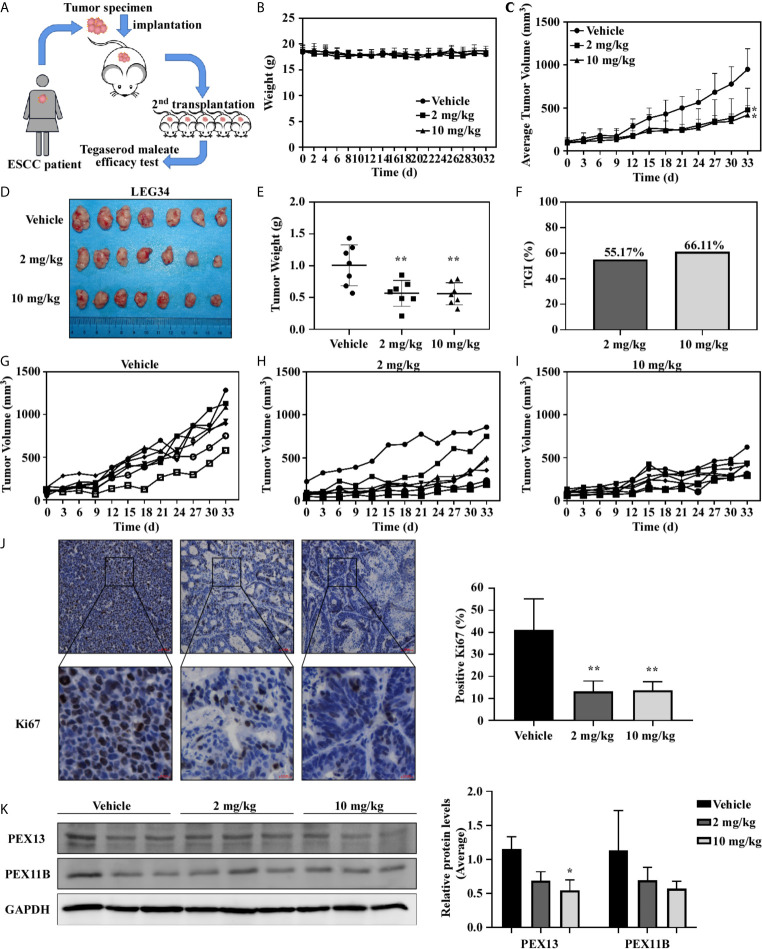
Tegaserod maleate inhibits the growth of ESCC PDX *in vivo*. **(A)** Schematic diagram to evaluate the effect of tegaserod maleate on tumor growth of ESCC PDX. **(B)** Tegaserod maleate has no toxic effects on the body weight of mice. Body weights were obtained from treated or untreated groups mice every two days. **(C)** The average tumor volume of each group was measured every three days. **(D)** The photograph shows tumors in PDX mice treated with vehicle or tegaserod maleate (2 mg/kg or 10 mg/kg). **(E)** On the 33rd day, the tumor weight was measured. The tumor volumes in tegaserod maleate-treated mice was significantly reduced (**p < 0.01). **(F)** The tumor growth inhibition (TGI) with 2 mg/kg and 10 mg/kg tegaserod maleate treatment. **(G)** Growth curves of individual tumors in the vehicle group, **(H)** 2 mg/kg tegaserod maleate treated group and **(I)** 10 mg/kg tegaserod maleate treated group. **(J)** Immunohistochemical staining with Ki67 of tumor tissues in treated or untreated groups (left panel). The number of Ki67 staining positive cells was counted from the immunohistochemical results (right panel) (n = 3, **p < 0.01). **(K)** The protein levels of PEX13 and PEX11B in tumor tissues were detected by western blotting. Image J software program was used to measure band density. The average relative protein levels of PEX13 and PEX11B are shown (*p < 0.05).

The PDX model was used to further evaluate the antitumor activity of tegaserod maleate *in vivo*. A ESCC patient’s tumor (female, moderately differentiated squamous cell carcinoma of origin, which was named LEG34) tissue was implanted into the back of SCID (severe combined immunodeficiency) mice, thereafter, tegaserod maleate (2 or 10 mg/kg) was administered to the mice daily by gavage (**Figure 6A**). Compared with the vehicle group, treatment with 2 or 10 mg/kg tegaserod maleate had no effect on the body weight of the mice (**Figure 6B**) but significantly inhibited tumor growth (**Figures 6C, D**). We calculated tumor weight (**Figure 6E**) and found that tumor growth inhibition (TGI) reached 55.17% with 2 mg/kg and 66.11% with 10 mg/kg tegaserod maleate (**Figure 6F**). Subsequently, we observed the growth of individual tumors in the different groups (vehicle, 2, or 10 mg/kg tegaserod maleate), and found that tegaserod maleate inhibited tumor growth (**Figures 6G-I**). We used immunohistochemistry to analyze the expression of Ki67 in tumor tissues (**Figure 6J**). Simultaneously, we detected the expression of PEX11B, PEX13 (**Figure 6K**) and PMP70 (**Figure S5B**) in tumor tissues by western blotting. Peroxisome and expression of all these proteins was suppressed in the tegaserod maleate-treatment group compared with that in the vehicle group.

### The Molecular Mechanism of Tegaserod Maleate Inhibition of ESCC

**Figure 7:**
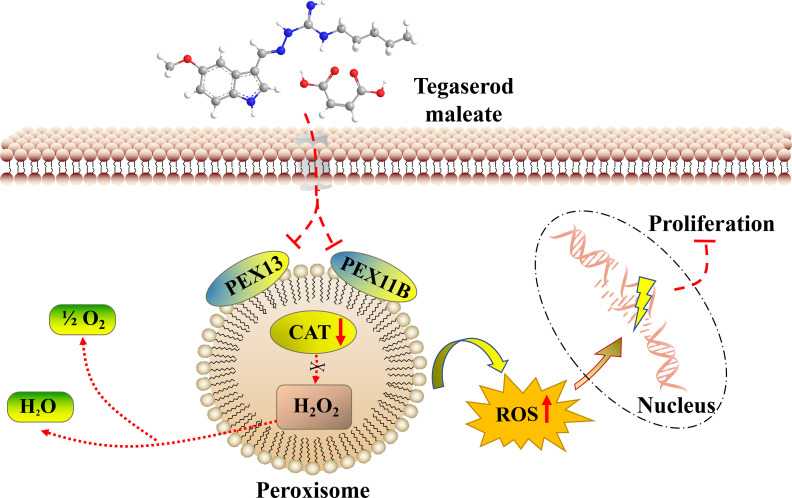
The molecular mechanism diagram of tegaserod maleate inhibition of ESCC.

Through the above studies, we believe that tegaserod maleate restricts the function of peroxisomes by downregulating PEX11B and PEX13 in the peroxisome signaling pathway. Weakened catalase is unable to remove hydrogen peroxide in the peroxisome, resulting in the increase of ROS in cells (**Figure 7**). Therefore, tegaserod maleate could inhibit ESCC proliferation both *in vitro* and *in vivo*.

## Discussion

EC is a digestive tract malignant tumor that currently ranks sixth in incidence and fourth in mortality in China (18). Currently, there are no effective targeted drugs for the treatment of EC and prevention. It is advantageous to find a new anticancer use among existing drugs that may already have an established safety profile (19). NSAIDS, such as aspirin, have been found lower risk of cholangiocarcinoma and colorectal cancer (20, 21). Not only that, but there is a strong interest in reusing metformin to prevent cancer and cancer recurrence (22). Our recently study indicated atorvastatin, a lipid-lowering drug (23), and mefloquine, an anti-malarial drug (24), had a strong inhibitory effects on ESCC proliferation. Here, we screened the FDA-approved drug and found tegaserod maleate could inhibit proliferation of ESCC both *in vivo* and *in vitro*. We believe that tegaserod maleate has the potential to positively influence ESCC treatment or recurrence prevention.

Tegaserod maleate is used for the treatment of constipation-type irritable bowel syndrome as a 5-hydroxytryptamine 4-receptor partial agonist (25). Studies have shown that 5-hydroxytryptamine plays a mitogenic role in colon cancer cells, and 5-hydroxytryptamine 4-receptor is significantly expressed in both colon cancer tissue and cells (26). Tegaserod maleate has been shown to exert anticancer activity by inducing apoptosis in melanoma cells (27), and it has been identified as a JAK/STAT3 signal inhibitor, impeding the growth of a variety of cancer cells, including lung cancer, prostate cancer, colon cancer, and cervical cancer (28). JAK/STAT3 pathway also plays an important role in cell proliferation. Here, we found that tegaserod maleate could inhibit ESCC proliferation. Through proteomics analysis, we found that the antitumor mechanism of tegaserod maleate is through peroxisome pathway inhibition. Studies have also shown that the STAT3 inhibitor niacinamide could rescue hepatitis C virus-induced inhibition of peroxisomal genes (29). All these results suggest tegaserod maleate can suppress cell proliferation through both JAK/STAT3 pathway and peroxisome pathway.

Peroxisomes are organelles that contain a variety of enzymes and that scavenge ROS (30). At the molecular level, catalase activity decreased in a dose-dependent manner in ESCC cell lines after tegaserod maleaste treatment, and the fluorescence intensity of ROS increased. Tegaserod maleate produces the above results that impedes the function of peroxisomes by inhibiting the key molecules PEX11B and PEX13. PEX11B participates in the proliferation and division of the peroxisome itself (31), whereas PEX13 acts as a docking factor for the transfer of matrix proteins into the peroxisome body (32). Both are highly expressed in ESCC and closely related to the poor prognosis of ESCC. These data provide strong evidence for the therapeutic potential of tegaserod maleate to inhibit ESCC.

In summary, our data provide evidences that tegaserod maleate inhibits the proliferation of ESCC by impeding peroxisome function. In mechanistic terms, tegaserod maleate reduces proteins levels of the peroxisome membrane proteins PEX11B and PEX13, affecting the function of peroxisomes and achieving tumor suppression. Our findings highlight the new use of tegaserod maleate in ESCC treatment or prevention.

## Data Availability Statement

The raw data supporting the conclusions of this article will be made available by the authors, without undue reservation.

## Ethics Statement

The animal study was reviewed and approved by Research Ethics Committee of Zhengzhou University.

## Author Contributions

KL and ZD conceived the study. XW completed most of the experiments and wrote the manuscript. YJ proofread the manuscript. ZW and YW participated in cell assays. HZ and YG made bioinformatics analysis. AL and ZB did immunohistochemistry. DW and XC performed animal assays. JZ supervised the project. ZD and KL approved the final manuscript. All authors contributed to the article and approved the submitted version.

## Funding

This research was supported by the National Science & Technology Major Project Key New Drug Creation and Manufacturing Program, China (No. 2018ZX09711002), National Natural Science Foundations of China (grant number 81872335), National Natural Science Youth Foundation (grant number 81902486), the Natural Science Foundation of Henan (grant number 161100510300), the Science and Technology Project of Henan Province (grant number 212102310187).

## Conflict of Interest

The authors declare that the research was conducted in the absence of any commercial or financial relationships that could be construed as a potential conflict of interest.

## Publisher’s Note

All claims expressed in this article are solely those of the authors and do not necessarily represent those of their affiliated organizations, or those of the publisher, the editors and the reviewers. Any product that may be evaluated in this article, or claim that may be made by its manufacturer, is not guaranteed or endorsed by the publisher.

## References

[1] Sung H Ferlay J Siegel RL Laversanne M Soerjomataram I Jemal A. Global Cancer Statistics 2020: GLOBOCAN Estimates of Incidence and Mortality Worldwide for 36 Cancers in 185 Countries. CA Cancer J Clin (2021) 71(3):209–249. 10.3322/caac.21660 33538338

[2] Huang J Koulaouzidis A Marlicz W Lok V Chu C Ngai CH. Global Burden, Risk Factors, and Trends of Esophageal Cancer: An Analysis of Cancer Registries From 48 Countries. Cancers (Basel) (2021) 13(1):141. 10.3390/cancers13010141 PMC7795486 33466239

[3] Pennathur A Gibson MK Jobe BA Luketich JD. Oesophageal Carcinoma. Lancet (2013) 381(9864):400–12. 10.1016/S0140-6736(12)60643-6 23374478

[4] Ashburn TT Thor KB. Drug Repositioning: Identifying and Developing New Uses for Existing Drugs. Nat Rev Drug Discovery (2004) 3(8):673–83. 10.1038/nrd1468 15286734

[5] Xie Y Yao K Dong Z Liu K. Targeting Nutrient Metabolism With FDA-Approved Drugs for Cancer Chemoprevention: Drugs and Mechanisms. Cancer Lett (2021) 510:1–12. 10.1016/j.canlet.2021.03.029 33857528

[6] Marciniak TA Serebruany V. Should We Use Tegaserod for Irritable Bowel Syndrome? Am J Ther (2019) 26(3):e417–20. 10.1097/MJT.0000000000000947 30946048

[7] Wanders RJ Waterham HR. Biochemistry of Mammalian Peroxisomes Revisited. Annu Rev Biochem (2006) 75:295–332. 10.1146/annurev.biochem.74.082803.133329 16756494

[8] Goldfischer S Moore CL Johnson AB Spiro AJ Valsamis MP Wisniewski HK. Peroxisomal and Mitochondrial Defects in the Cerebro-Hepato-Renal Syndrome. Science (1973) 182(4107):62–4. 10.1126/science.182.4107.62 4730055

[9] Glorieux C Calderon PB. Catalase, a Remarkable Enzyme: Targeting the Oldest Antioxidant Enzyme to Find a New Cancer Treatment Approach. Biol Chem (2017) 398(10):1095–108. 10.1515/hsz-2017-0131 28384098

[10] Lismont C Nordgren M Van Veldhoven PP Fransen M. Redox Interplay Between Mitochondria and Peroxisomes. Front Cell Dev Biol (2015) 3:35. 10.3389/fcell.2015.00035 26075204 PMC4444963

[11] Xu Z Asahchop EL Branton WG Gelman BB Power C Hobman TC. MicroRNAs Upregulated During HIV Infection Target Peroxisome Biogenesis Factors: Implications for Virus Biology, Disease Mechanisms and Neuropathology. PloS Pathog (2017) 13(6):e1006360. 10.1371/journal.ppat.1006360 28594894 PMC5464672

[12] Montague TG Cruz JM Gagnon JA Church GM Valen E. CHOPCHOP: A CRISPR/Cas9 and TALEN Web Tool for Genome Editing. Nucleic Acids Res (2014) 42(Issue W1):W401–7. 10.1093/nar/gku410 PMC4086086 24861617

[13] Agrawal G Subramani S. De Novo Peroxisome Biogenesis: Evolving Concepts and Conundrums. Biochim Biophys Acta (2016) 1863(5):892–901. 10.1016/j.bbamcr.2015.09.014 26381541 PMC4791208

[14] Schrader M Bonekamp NA Islinger M. Fission and Proliferation of Peroxisomes. Biochim Biophys Acta (2012) 1822(9):1343–57. 10.1016/j.bbadis.2011.12.014 22240198

[15] Emmanouilidis L Gopalswamy M Passon DM Wilmanns M Sattler M. Structural Biology of the Import Pathways of Peroxisomal Matrix Proteins. Biochim Biophys Acta (2016) 1863(5):804–13. 10.1016/j.bbamcr.2015.09.034 26450166

[16] Chandrashekar DS Bashel B Balasubramanya S Creighton CJ Ponce-Rodriguez I Chakravarthi B. UALCAN: A Portal for Facilitating Tumor Subgroup Gene Expression and Survival Analyses. Neoplasia (2017) 19(8):649–58. 10.1016/j.neo.2017.05.002 PMC5516091 28732212

[17] Nagy A Lanczky A Menyhart O Gyorffy B. Validation of miRNA Prognostic Power in Hepatocellular Carcinoma Using Expression Data of Independent Datasets. Sci Rep (2018) 8(1):9227. 10.1038/s41598-018-27521-y 29907753 PMC6003936

[18] Cao J Xu H Li W Guo Z Lin Y Shi Y. Nutritional Assessment and Risk Factors Associated to Malnutrition in Patients With Esophageal Cancer. Curr Probl Cancer (2021) 45(1):100638. 10.1016/j.currproblcancer.2020.100638 32829957

[19] Rayan A Raiyn J Falah M. Nature is the Best Source of Anticancer Drugs: Indexing Natural Products for Their Anticancer Bioactivity. PloS One (2017) 12(11):e187925. 10.1371/journal.pone.0187925 PMC5679595 29121120

[20] Shen X Shen X. A Potential Role for Aspirin in the Prevention and Treatment of Cholangiocarcinoma. Int J Cancer (2021) 148(6):1323–30. 10.1002/ijc.33323 32997790

[21] Grancher A Michel P Di Fiore F Sefrioui D. Aspirin and Colorectal Cancer. Bull Cancer (2018) 105(2):171–80. 10.1016/j.bulcan.2017.09.013 29153543

[22] Heckman-Stoddard BM DeCensi A Sahasrabuddhe VV Ford LG. Repurposing Metformin for the Prevention of Cancer and Cancer Recurrence. Diabetologia (2017) 60(9):1639–47. 10.1007/s00125-017-4372-6 PMC5709147 28776080

[23] Yuan Q Dong CD Ge Y Chen X Li Z Li X. Proteome and Phosphoproteome Reveal Mechanisms of Action of Atorvastatin Against Esophageal Squamous Cell Carcinoma. Aging (Albany NY) (2019) 11(21):9530–43. 10.18632/aging.102402 PMC6874460 31697643

[24] Xie Y Zhang J Lu B Bao Z Zhao J Lu X. Mefloquine Inhibits Esophageal Squamous Cell Carcinoma Tumor Growth by Inducing Mitochondrial Autophagy. Front Oncol (2020) 10:1217. 10.3389/fonc.2020.01217 32850358 PMC7400730

[25] Evans BW Clark WK Moore DJ Whorwell PJ. Tegaserod for the Treatment of Irritable Bowel Syndrome and Chronic Constipation. Cochrane Database Syst Rev (2007) 4):D3960. 10.1002/14651858.CD003960.pub3 17943807

[26] Ataee R Ajdary S Rezayat M Shokrgozar MA Shahriari S Zarrindast MR. Study of 5HT3 and HT4 Receptor Expression in HT29 Cell Line and Human Colon Adenocarcinoma Tissues. Arch Iran Med (2010) 13(2):120–5. 10.4103/0256-4947.60530 20187666

[27] Liu W Stachura P Xu HC Umesh GN Cox F Wang R. Repurposing the Serotonin Agonist Tegaserod as an Anticancer Agent in Melanoma: Molecular Mechanisms and Clinical Implications. J Exp Clin Cancer Res (2020) 39(1):38. 10.1186/s13046-020-1539-7 32085796 PMC7035645

[28] Zhang L Song Q Zhang X Li L Xu X Xu X. Zelnorm, an Agonist of 5-Hydroxytryptamine 4-Receptor, Acts as a Potential Antitumor Drug by Targeting JAK/STAT3 Signaling. Invest New Drugs (2020) 38(2):311–20. 10.1007/s10637-019-00790-8 31087223

[29] Lupberger J Croonenborghs T Roca SA Van Renne N Juhling F Oudot MA. Combined Analysis of Metabolomes, Proteomes, and Transcriptomes of Hepatitis C Virus-Infected Cells and Liver to Identify Pathways Associated With Disease Development. Gastroenterology (2019) 157(2):537–51. 10.1053/j.gastro.2019.04.003 PMC8318381 30978357

[30] Deori NM Kale A Maurya PK Nagotu S. Peroxisomes: Role in Cellular Ageing and Age Related Disorders. Biogerontology (2018) 19(5):303–24. 10.1007/s10522-018-9761-9 29968207

[31] Bonekamp NA Grille S Cardoso MJ Almeida M Aroso M Gomes S. Self-Interaction of Human Pex11pbeta During Peroxisomal Growth and Division. PloS One (2013) 8(1):e53424. 10.1371/journal.pone.0053424 23308220 PMC3538539

[32] Gould SJ Kalish JE Morrell JC Bjorkman J Urquhart AJ Crane DI. Pex13p is an SH3 Protein of the Peroxisome Membrane and a Docking Factor for the Predominantly Cytoplasmic PTs1 Receptor. J Cell Biol (1996) 135(1):85–95. 10.1083/jcb.135.1.85 8858165 PMC2121023

