# Supplementary material for: Tegaserod Maleate Inhibits Esophageal Squamous Cell Carcinoma Proliferation by Suppressing the Peroxisome Pathway
Source: Front Oncol. 2021 Aug 4;11:683241. doi: 10.3389/fonc.2021.683241 (PMC8372369; doi:10.3389/fonc.2021.683241)
Supplement: Supplementary file 1 [file DataSheet_1.docx]

Supplementary Material

## Supplementary Figures


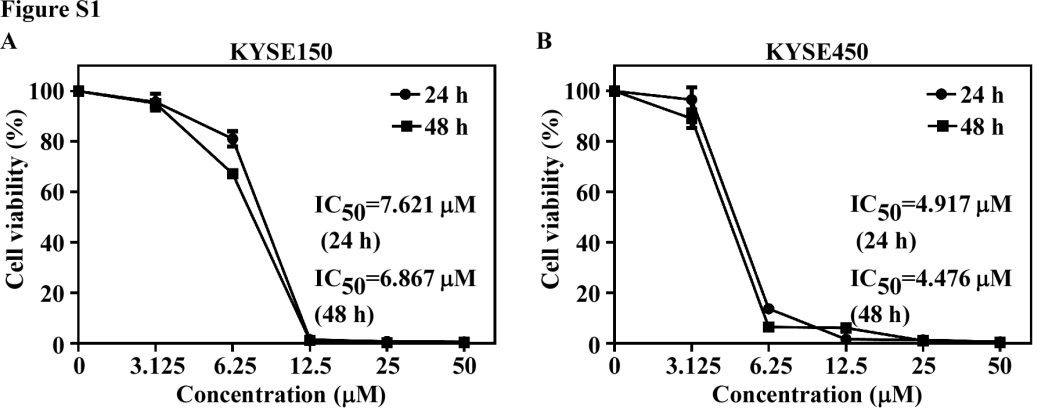


**Supplementary Figure 1. The curve graph of the cytotoxicity assay in KYSE150 (A) and KYSE450 cells (B).**


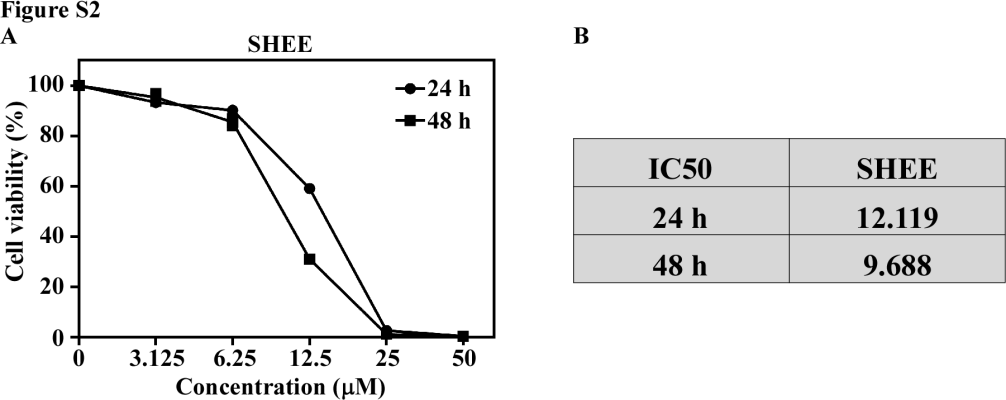


**Supplementary Figure 2. In accordance with the cytotoxicity assay results on normal esophageal epithelial cell line SHEE cells (A), the IC50 values of SHEE at 24 and 48 h were calculated (B).**


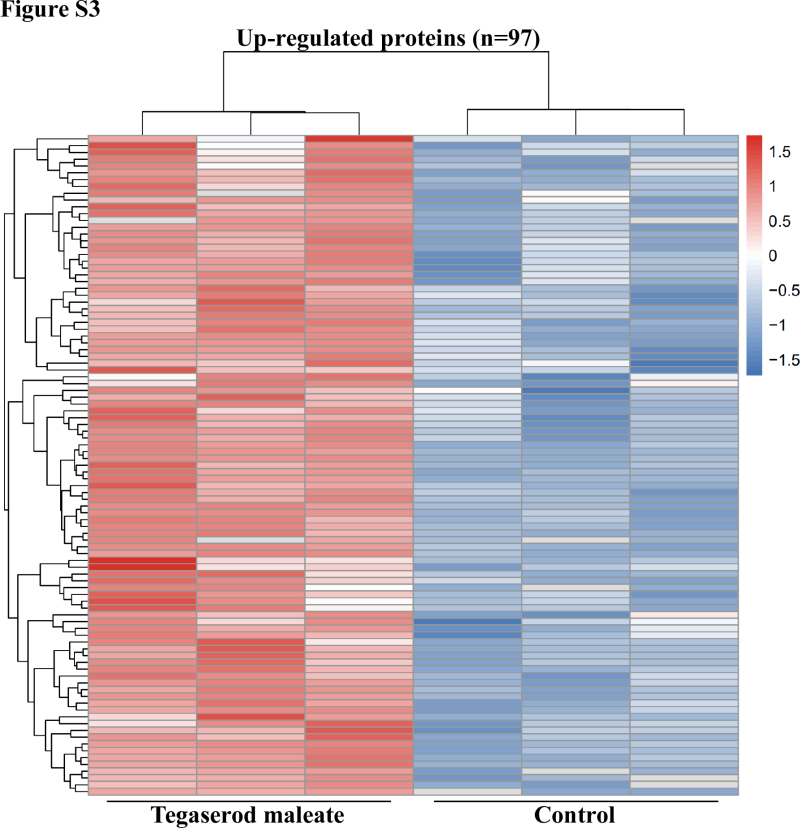


**Supplementary Figure 3. The heatmap of up-regulated proteins after normalization (n=97).**


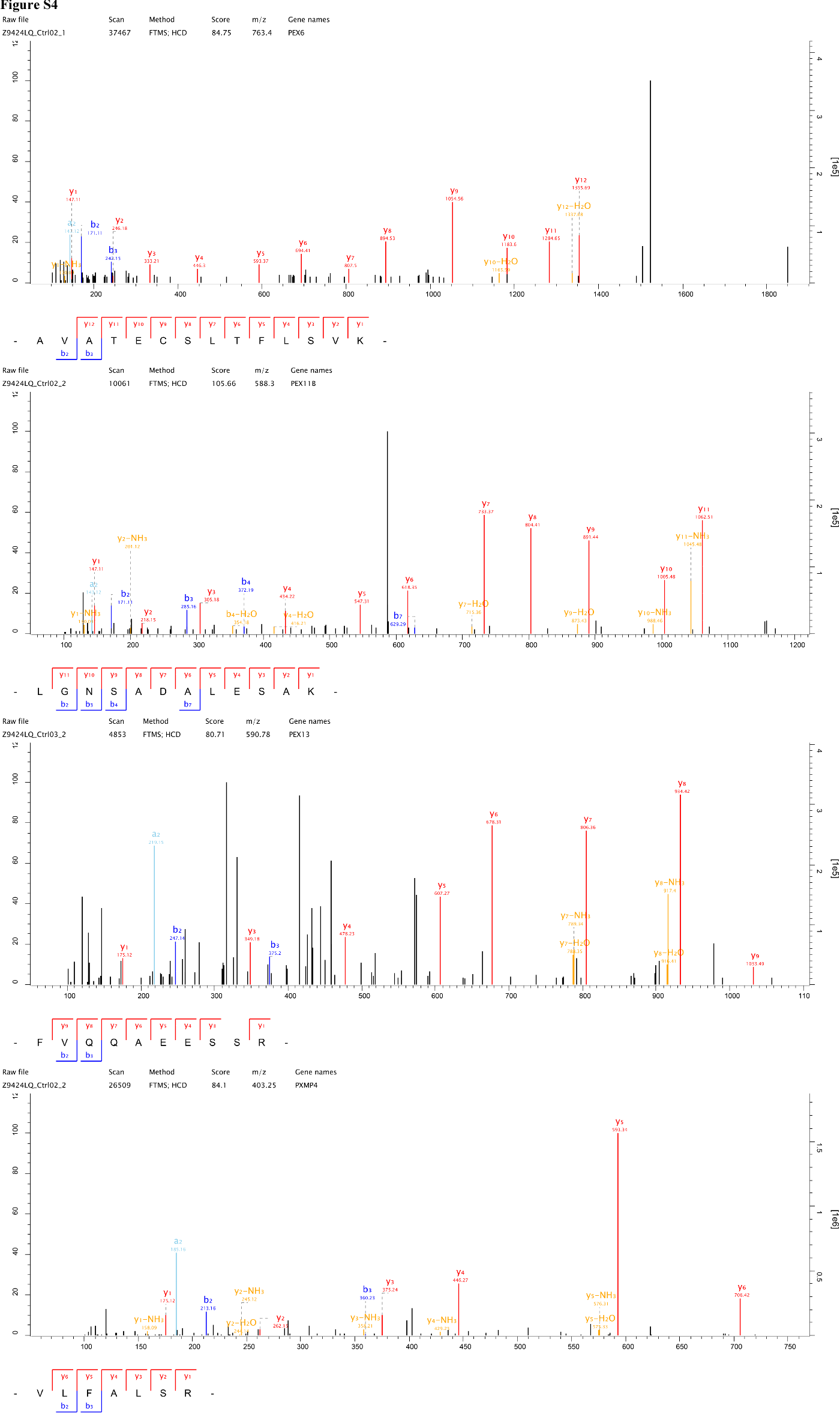


**Supplementary Figure 4. Peptide mass spectrum of PEX6, PEX11B, PEX13, and PXMP4 involved in peroxisome pathway. The spectra of these proteins were represented by the highest specific peptide score.**

**
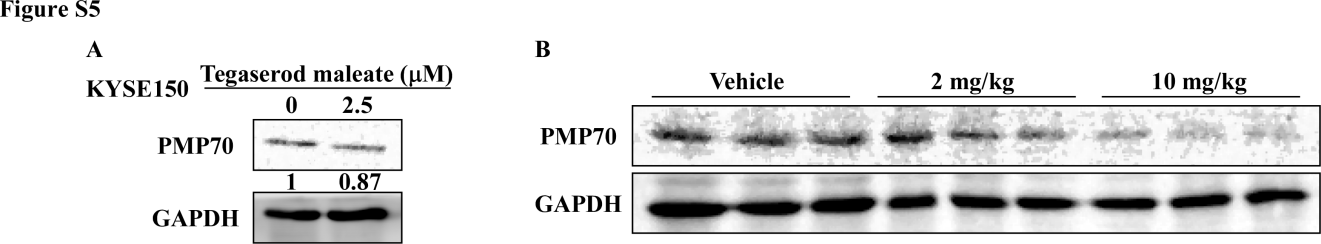
**

**Supplementary Figure 5. Western blotting showed the changes of peroxisomes after tegaserod maleate treatment. (A)** Tegaserod maleate down-regulates the protein level of PMP70 in KYSE150 cells. **(B)** The changes of peroxisome after tegaserod maleate treatment were detected by western blotting analysis of PMP70 in tumor tissues.

**
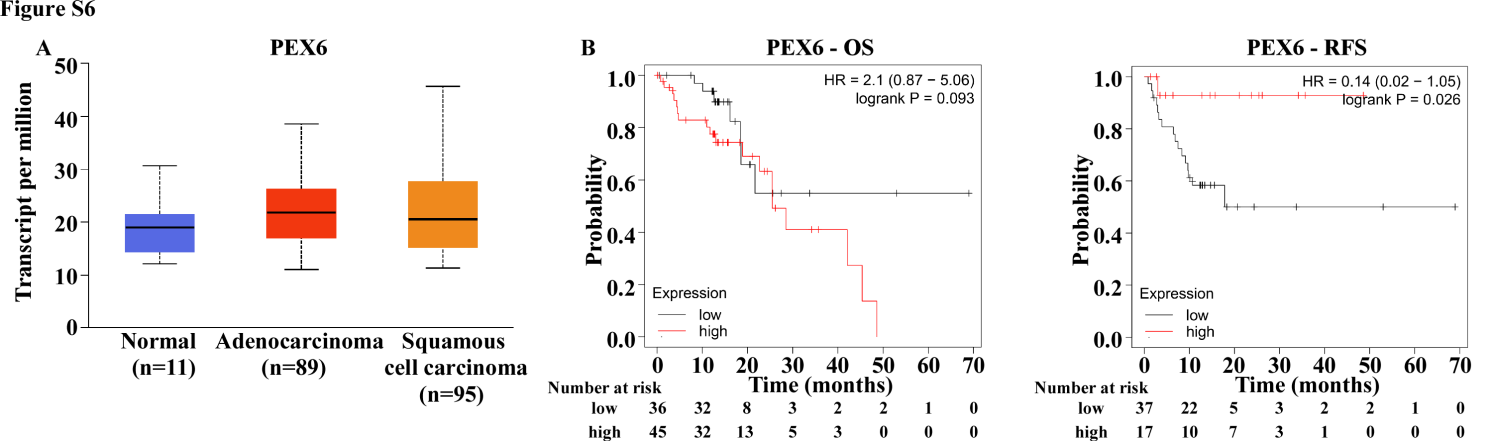
**

**Supplementary Figure 6. The expression and prognosis of PEX6 in ESCC patients. (A)** The expression of PEX6 in ESCC from the TCGA database in UALCAN. **(B)** Kaplan–Meier curves of OS and RFS in patients with PEX6.
